# Fast self-curing α-tricalcium phosphate/β-dicalcium silicate composites beneficial for root canal sealing treatment

**DOI:** 10.1016/j.heliyon.2022.e10713

**Published:** 2022-09-21

**Authors:** Youyang Zheng, Xianyan Yang, Shuxin Liu, Siqi Bao, Yuyue Xu, Yunyi Wang, Feng Zhang, Zhongru Gou

**Affiliations:** a Department of Stomatology, The Second Affiliated Hospital, School of Medicine Zhejiang University, Hangzhou 310009, China; b Bio-nanomaterials and Regenerative Medicine Research Division, Zhejiang-California International Nanosystems Institute, Zhejiang University, Hangzhou 310058, China; c School of Stomatology, Zhejiang University School of Medicine, Hangzhou 310006, China; d Department of Stomatology, The Children's Hospital, Zhejiang University School of Medicine, National Clinical Research Center for Child Health, Hangzhou 310003, China

**Keywords:** Setting time, Anti-microleakage, α-Tricalcium phosphate, β-Dicalcium silicate, Root canal sealing

## Abstract

**Objectives:**

α-tricalcium phosphate (α-TCP) and β-dicalcium silicate (β-C2S) have attracted much attention since these two types of self-curing Ca-phosphate and Ca-silicate are valuable biomaterials for bone defect or endodontic therapy. However, the injectable paste of their individual with high liquid/solid ratio is junior for root canal sealing due to very long self-setting time, low pH value and/or much volume shrinkage during paste-to-cement transformation.

**Methods:**

Our studies evaluated the effect of biphasic ratio, liquid/solid ratio and pH condition of aqueous medium on setting time and mechanical strength of this biphasic composite cement, and also the hydroxyapatite re-mineralization potential and anti-microleakage level of the cements with different α-TCP/β-C2S ratio were explored in vitro. A control group free of paste filler was included in the extracted teeth model. Dentine re-mineralization and microleakage degree were observed by scanning electron microscopy and microCT reconstruction analysis.

**Results:**

It indicated that the weak acidic solution with pH value of 6.0 may produce a significantly shorter initial setting time (from 90 min to less 20 min) and expected final setting time (<150 min) for the biphasic composite (2:1 or 1:2) in comparison with the pure β-C2S. Notably, the phasic composites exhibited limited microleakage and induced hydroxyapatite mineralization in the dentine tubules. These hydraulic pastes also produced strong alkaline feature and appreciable compressive resistance (12–18 MPa) after setting for a very short time stage. Moreover, a link between the addition of α-TCP leading to fast re-mineralization reaction was established.

**Significance:**

Our findings suggest that the appreciable self-setting and physicochemical properties adaption to root canal sealability make α-TCP/β-C2S composites as preferential candidates for endodontic treatments.

## 1 Introduction

Root canal treatment is a versatile approach to treat periradicular inflammation and reversible pulpitis in endodontics. It is well known that the clinical outcomes depend mainly on the thorough debridement and followed by full closure of the diseased root canal, even the dentine tubules. However, the inadequate obturation risks tissue fluid penetration to the root apex and subsequently leads to periradicular inflammation, and such serious consequence is the important concern for dentists [1, 2, 3].

In the past three decades, the antimicrobial calcium hydroxide (CH)-based suspension paste has often been used as the intracanal cleaning medicament to eliminate the micro-organisms. In particular, the high pH value may induce hard tissue formation during Ca^2+^ ions release in the root canal and thus induce the mineralized tissue deposition [4, 5]. In fact, such alkalic condition is also responsible for its bactericidal effect, accompanying with a pronounced destructive effect on the bacterial cell membranes [6]. Gomes et al. have found some anaerobic Gram-negative bacteria are significantly susceptible to CH pastes, and the microbial susceptibility, ranked from weakest to strongest, can be presented as E. faecalis, C. albicans, S. aureus, P. gingivalis, P. endodontalis and P. intermedia. Particularly, the last two microorganisms required only several minutes to be eliminated during in the antimicrobial activity test [7, 8]. Indeed, it is well agreed that, except for non-adherence to dentine and low sealing ability, the CH paste exhibits the delayed effect when using CH to induce hard tissues, and finally the dentin weakening effect of CH may lead to cervical root fractures in immature teeth [9]. Hence, the long-term stable filler should provide permanent apical sealing and even stimulate the periradicular tissue healing and/or re-mineralization of the dentine tubules. Unfortunately, the conventional composite resin and glass ionomer cements are potentially contradictive in biological effect in vivo, and even may cause long-term inflammatory responses [10, 11].

In general, the Ca-silicate-based inorganic pastes have gained clinical acceptance as valuable root canal fillers for nearly 30 years [12]. For instance, mineral trioxide aggregate (MTA) is composed of tricalcium silicate (C3S), β-dicalcium silicate (β-C2S) and other inorganic components. Previous studies have shown that MTA can prevent microleakage, and even promote the original tissue regeneration in the dental pulp tissues, whereas the clinical applications indicate that MTA is suboptimal for some clinical conditions due to its discoloration potential and long self-setting time [13, 14, 15]. In fact, the expected characteristics of sealing materials usually exhibit excellent hydroxyapatite (HA) re-mineralization potential to completely seal the root canal system, and good self-curing efficiency and long-term stability to convenient treatment and permanent anti-leakage, and finally inhibit bacteria penetration. In order to improving the antimicrobial properties, radio-opacity and biocompatibility of Ca-silicate fillers [16, 17], the C3S-based pastes have been investigated systematically in the past two decades. Zhou et al. have studied that Mg-phosphate cement could be acted as curing accelerator of the C3S-based premixed fillers [18]. Also, CaCl_2_ was suggested to add into the C3S cement for accelerating its curing and enhancing strength [19, 20]. Moreover, some studies including ourselves have also found that introduction of other inorganic slats or foreign ion dopants may enhance antibacterial potential or accelerate the self-curing process of the Ca-silicate pastes [21, 22, 23, 24]. In general, the Ca-silicate cement pastes set through a hydration reaction after mixing with aqueous solution. Various hydration products form during the reaction, namely different phases of calcium silicate hydrate (CSH) as colloidal CSH gel and radial acicular CSH crystals. However, a long time stage (several hours) is usually required for the conversion from CSH gel to hardening phase formation and the complete hydration reaction even requires several days [15]. Accordingly, the hydration process of Ca-silicate pastes is mainly dependent on the dissolution-cohesion reaction, yet its long curing time is not resolved thoroughly.

Recently, some calcium phosphate (CaP) materials have attracted more attention in orthopaedics because of their excellent and inherent self-setting property. For instance, α-tricalcium phosphate (α-TCP) and related cements have been developed as bone defect or root canal fillers [25, 26, 27]. The conversion of α-TCP into more stable CaP phase (so-called calcium-deficient HA) has enjoyed a longer history of clinical use for bone augmentation because of its fast self-setting feature [28, 29]. The α-TCP powders may readily react with water and the particles could grow as a mass of interlocking aggregates that provide the strength of hydrating cement. Nevertheless, the disadvantages including poor re-mineralization and appreciable volume shrinkage during slurry-to-cement transformation may affect its applications in root canal treatment.

Herein, we hypothesized that addition of α-TCP is able to tune the self-curing progress of β-C2S-based pastes. In fact, the former showed very fast setting and hydraulic properties in weak acidic solution, but in contrast, the β-C2S cement shows appreciable late-stage hydraulic properties in vitro. Hence, the fabrication of the α-TCP/β-C2S composite pastes with appropriate aqueous solution can be used potentially in root canal filling treatment. In this study, the setting time of the cement pastes with varying α-TCP/β-C2S ratio were investigated, and then the potential effect of pH level in the aqueous solution on the setting time and mechanical strength were evaluated. Also, the surface reactivity and cement-dentine integration ex vivo were studied. The investigations of composition–setting–microleakage relationship of single-phase and biphasic paste setting process have been performed to explore the main property refinement in endodontic application areas.

## 2 Materials and methods

### 2.1 Materials

The analytic reagents including trishydroxymethyl aminomethane (Tris), ammonia (NH_3_·H_2_O, ∼28 wt.%), calcium nitrate (Ca(NO_3_)_2_·4H_2_O), tertraethyl orthosilicate (TEOS), diammonium phosphate ((NH_4_)_2_HPO_4_), acetic acid, citric acid, glycol and absolute ethanol were purchased from Sinopharm Chemical Reagent Co. China. Artificial saliva (ISO/TR10271) and attapulgite (ATT) nanofibers were bought from Shanghai Yuanye Bio-Technology Co. and Mingguang Mingjiu S&T Co., China, respectively. The chemicals were used directly without any purification pre-treatment.

### 2.2 Powder preparation

The pure α-TCP powder were prepared via a wet-chemical process as described elsewhere [30] and followed by high-temperature calcining at 1160 °C for 2 h. The β-C2S powders were prepared by a sol-gel method based on our previous study [23]. Briefly, calcium nitrate, TEOS, and acetic acid were added into ethanol-water mixture under magnetic stirring. The sol with Ca/Si ratio of 1.8 was aged at 60 °C for over 2 days and then dried at 80 °C overnight. The Ca/Si ratio below that in the stoichiometric β-C2S is thought to be favorable for controlling the free CaO content in the synthesized β-C2S powders [23]. The dried gels were calcined at 850 °C for 2 h, and then the free CaO (*f*_CaO_) content was measured according to the previous method [31]. Finally, the calcined α-TCP and β-C2S powders (45 g; ×4) were planetary ball-milled for 4 h (400 RPM; ∼22 °C) in the absolute ethanol medium (100 ml), respectively. The ball mass-to-powder ratio was 3:1, and the diameter of ZrO_2_ balls were 2.4 mm and 7.2 mm (1:1 in mass), respectively. The ball-milled superfine powders were then dried at 80 °C overnight, and then characterized by scanning electron microscope (SEM, HITACHI, S4800; Japan) and X-ray diffraction (XRD, Rigaku; Japan), respectively.

### 2.3 Paste preparation

The solid powders were firstly prepared by mixing α-TCP and β-C2S powders with mass ratio of 3:0, 2:1, 1:2, and 0:3, respectively. The particle size of the superfine powders was determined by laser granulometry on a zetasizer nano (Malvern, S90). The mixed powders (20.0 g) and ATT nanofibers (0.20 g) were each weighted to obtain as cement powders and ground for 5 min in a planetary ball mill for better homogeneity. The ATT nanofiber (∼2 μm in length) was used as a potential aid for enhancing structural densification of the self-cured cement. Then, the 2% CaCl_2_ solution (pH ∼7.8) was added dropwise in the biphasic powders with a liquid-to-solid (*L*/*S*) ratio of 0.7, 0.8, and 0.9 ml g^−1^, respectively. Also, the pH value (9.0, 7.4, 6.0) in CaCl_2_ solution was adjusted and balanced with dilute Tris or citric acid solution and the different groups of paste were prepared. The pastes were stirred for 45 s, transferred to plastic molds (Ø6.0 × 8.0 mm) and stored in a 100% humidity water bath at 37 °C. The samples with different TCP/C2S ratios (3:0, 2:1, 1:2, 0:3) were denoted as TCP3/C2S0, TCP2/C2S1, TCP1/C2S2, and TCP3/C2S0, respectively.

### 2.4 Setting time

The initial setting time (*IST*) and final setting time (*FST*) of the fresh paste samples were measured by Gillmore penetrometer (initial needle ∅ = 2.12 mm & final needle ∅ = 1.06 mm) according to according to the ASTM standard C-266 as reported in our previous studies [32]. Briefly, the experiments were carried out on the as-prepared pastes via mixing 5.0 g of powders with different volumes of solution for 30 s. The paste was immediately cast in the stainless steel mold with a diameter of 15 mm and a height of 20 mm, which were then cured in water-bath in controlled temperature and humidity conditions (37 °C; RH ≥ 95%). To find the two parameters (*IST* and *FST*), the samples were removed from the incubation chamber and measured by the with Gillmore penetrometer every 2 min. The time from the inorganic pastes being cured at 37 °C to the setting point was used as the setting times. Each group was repeated four times, and the average value was calculated.

### 2.5 Injectability of the pastes

The injectability of cement pastes was estimated from the samples that could be injected using a 5.0 mm inner diameter, 5 ml syringe, with needle of 20G gauge size (Ø 0.60 mm). Firstly, 2.0 g of paste was loaded into the syringe and mechanically injected at a constant pressure of 6.8 bar. Injection operation was carried out until the paste could not be extruded. The mass of transferred paste and the mass remaining in the syringe were weighed and the percentage calculated against time. Four specimens were measured for each group, and the average value was calculated.

### 2.6 pH variation in vitro

The pH variation of the cements (Ø 6 × 4 mm) after self-curing for 12 h were estimated in 0.05 mol l^−1^ Tris buffer (pH 7.4) at 37 °C with a surface area-to-volume ratio of 0.1 cm^−1^. The 25 vol.% aqueous buffer was refreshed every 24 h. At the pre-set time point from 5 min to 120 h, the pH variation of the cement bed by contacting the electrode head during immersion was measured. Finally, the cement samples were removed from the buffer, gently rinsed with absolute ethanol, and dried (85 °C) up to mass constancy before weighing. The changes in mass were expressed as the percentage of the initial mass.

### 2.7 Mechanical strength analysis

As for compressive mechanical test, the cement specimens (Ø 6 × 6 mm) were prepared in a stainless steel mold and a pressure of 2 MPa was used to expel the potential microscopic air bubbles in the cement paste, and then stored in 100% humidity at 37 °C for a given interval (12, 24, and 120 h) in advance. The mechanical test was conducted on a universal testing machine (Instron) until failure at a loading rate of 0.5 mm/min to obtain compressive strength. The quantitative results were expressed as mean ± standard deviation (SD). Also, the 120- hour-set cement samples (Ø 6 × 6 mm) were characterized by XRD analysis.

### 2.8 Re-mineralization reaction in vitro

The simulated saliva was prepared by inorganic salts and urea with ion concentration nearly equal to that of human saliva (NaCl, 0.400 g l^−1^; KCl, 0.400 g l^−1^; CaCl_2_·6H_2_O, 0.795 g l^−1^; NaS·2H_2_O, 0.005 g l^−1^; Na_2_H_2_PO_4_·2H_2_O, 0.780 g l^−1^; urea, 1.000 g l^−1^). The 12-hours-set cement discs (Ø 6 × 2 mm) were immersed in the simulated saliva with a solid surface area-to-liquid volume ratio of 2:1 (mm^2^/ml). After immersion for 3 and 7 days, the cement specimens were gently rinsed with ethanol and dried in vacuum overnight for SEM observation.

In order to characterize the apatite-mineralization ability in vitro in root canal system, the external calculus and tissue debris were carefully removed with a scalpel blade from the human freshly extracted teeth with mature apices and single canal configuration. The extracted teeth were obtained from the Second Affiliated Hospital, School of Medicine of Zhejiang University, according to standard guidelines approved by the Zhejiang University Ethics Committee (I2022521). Then, the teeth were radiographed to confirm the existence of straight canals and were decoronated to a standardized root length of 14 mm from apex. The teeth were divided into four groups of 4 canals, and filled with one of the pastes. Additionally, the teeth without filling paste were used as control. Then, the samples were stored in a 37 °C water-bath with saturation humidity for 48 h and followed by immersing in simulated saliva (10 ml) at 37 °C with for 5 days.

### 2.9 Endodontic space sealing ex vivo

Human freshly extracted teeth free of crack, single-rooted, were used in this study. As described in our previous study [23], the four groups of paste-filled teeth were stored in 100% humidity at 37 °C for 48 h and they were again covered with a new layer of nail varnish leaving only the apex (∼1 mm) free for penetration. Four teeth without filler were also sealed externally with nail vanish as positive control. The specimens were stored in 100% relative humidity for 12 h and then immersed in 0.5% Rhodamine B at 37 °C for 3 days, using vacuum in the first 10 min. To highlight the dye penetration, cross-sections were made longitudinally with a low speed carborundum disc.

### 2.10 Statistical analysis

Experimental results were expressed as means ± standard difference. Statistical analysis was carried out using one-way ANOVA, and a *p*-value of less than 0.05 was considered statistically significant.

## 3 Results

### 3.1 Physicochemical characterization of the powders

**Figure 1:**
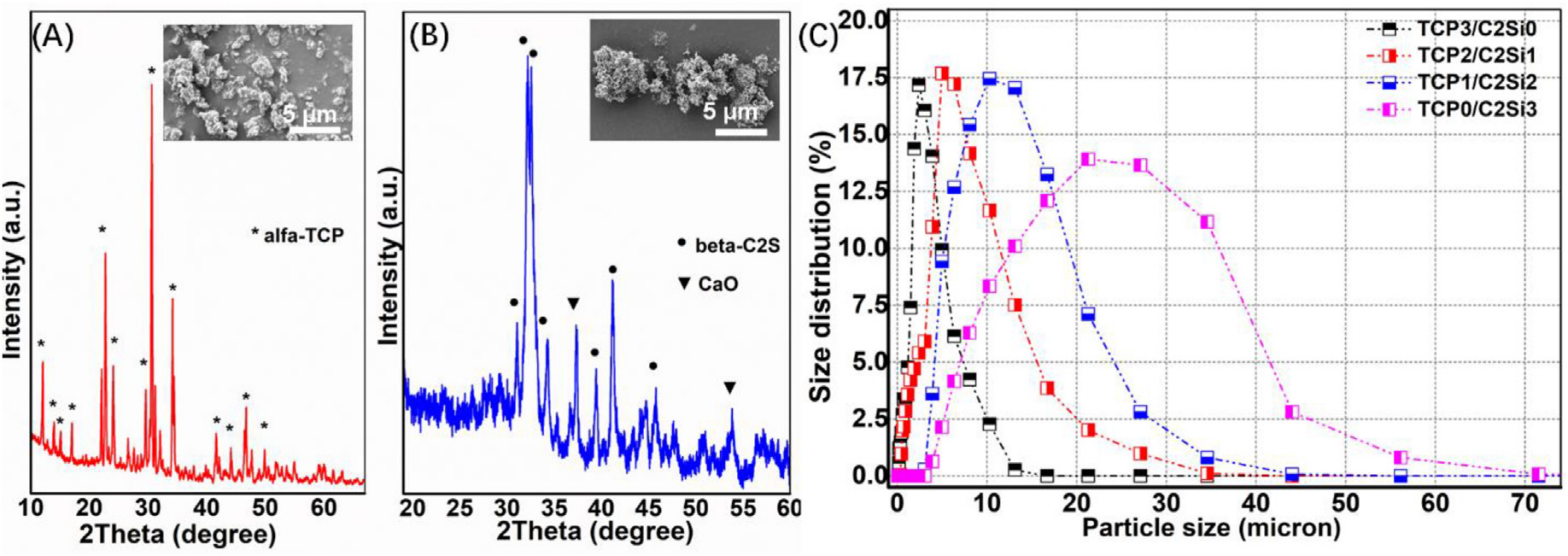
XRD patterns of α-TCP powder (A) and β-C2S powder (B) and particle size distribution (C) of the mixed superfine powders.

The phase compositions of the α-TCP and β-C2S powders were confirmed by XRD characterization, as shown in Figure 1. The pure α phase of TCP could be prepared by a facile wet-chemical route (Figure 1A), but the XRD patterns (34.8^o^, 53.7^o^/2θ) for the CaO phase may also be determined in the β-C2S powders (Figure 1B). The quantitative measurement (*f*_CaO_) indicated that there was 5.8 wt% free CaO in the powders. The SEM micrographs (Figure 1 A&B, inset) showed that the α-TCP and β-C2S samples exhibited irregular aggregation and the powders were nanoscale to microscopic particles. The particle size distribution analysis also confirmed that the particle sizes were increased with the increase of C2S content (Figure 1C).

### 3.2 Effect of powder compositions on setting properties

**Figure 2:**
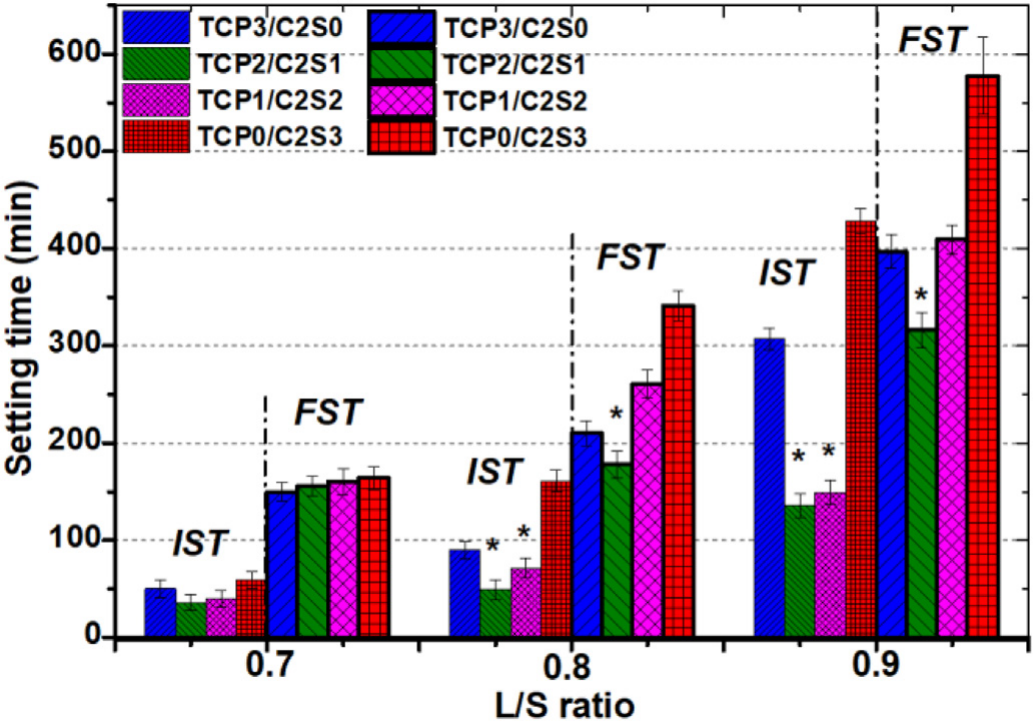
Initial setting time (*IST*) and final setting time (*FST*) of the TCP/C2S mixture paste prepared with the CaCl_2_ solution under different L/S ratio conditions. ∗*p* < 0.05 in comparison with pure TCP or C2S group.

Figure 2 showed the changes of the IST and FST values with different L/S ratio of the paste by using the CaCl_2_ solution as liquid phase. At the low L/S ratio condition of 0.7, the TCP3/C2S0 and TCP0/C2S3 pastes displayed a longer IST value (∼53–59 min) but the TCP2/C2S1 and TCP1/C2S2 pastes showed shortest IST value (<40 min). However, the four groups of samples showed very close FST values (150–164 min). With increasing respectively the L/S ratio up to 0.8 and 0.9, the IST and FST values were all increased. However, the biphasic composites of TCP2/C2S1 and TCP1/C2S2 showed very lower IST value in comparison with the other pure α-TCP (TCP3/C2S0) or pure β-C2S (TCP0/C2S3) paste. The FST value for the TCP2/C2S1 was significantly lower than the other three groups of samples at the L/S ratio of 0.8 and 0.9. It indicates that the setting time of pure α-TCP and β-C2S powders are more sensitive to the L/S ratio in comparison with the TCP−C2S composite.

All four groups of powder compositions at L/S ratio of 0.8 and 0.9 could be injected through the 20 G needle more than 80% and 95% of the pastes within 5 min and 8 min, respectively, yet less than 50% of paste at L/S of 0.7 for the 20G needle (Data not shown). With increasing β-C2S content up to 66%–100% (i.e. TCP1/C2S2 and TCP0/C2S3) at L/S of 0.7, the rapid decay in injectability was occurred. In order to achieve the desired setting qualities in the finished cements, the L/S ratio of 0.8 was selected as the optimal value in the following studies.

### 3.3 Effect of pH value of liquid medium on setting properties

**Figure 3:**
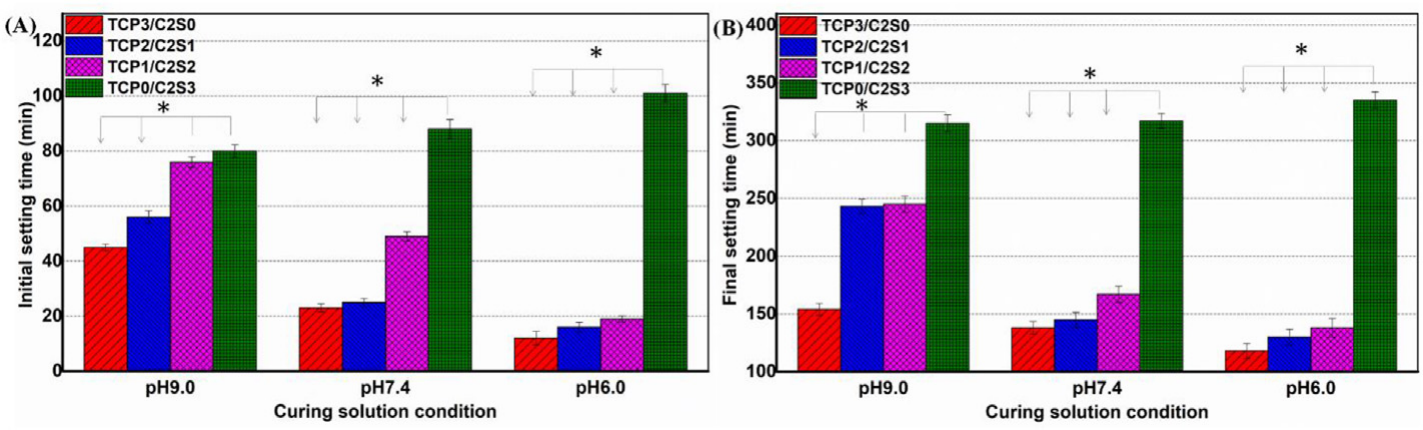
Initial setting time (A) and final setting time (B) of the TCP/C2S mixture paste prepared with weak alkaline, neutral, or acidic solutions with different pH values. ∗*p* < 0.05.

Taking into account the potential influence of the solution pH on setting time of the cement paste, we also measured the setting properties of the pastes prepared with the aqueous solutions with different pH values. In comparison with the neutral and acidic solution conditions, it was found from Figure 3 that the weak alkaline medium (pH ∼9.0) led to much longer IST and FST for the α-TCP-containing pastes. Meanwhile, the pastes also showed faster self-curing reaction with the increase of α-TCP content. When the weak acidic aqueous CaCl_2_ solution was used as the liquid phase, it was observed that all of α-TCP-containing pastes attained the IST values within 20 min, irrespectively of the amount of α-TCP in the pastes (Figure 3A). Evidently, the biphasic composites also had significantly shortened FST (∼130–140 min), which was reduced from around 250 min as the pH value was decreased from 9.0 to 6.0 (Figure 3B). In comparison, the TCP0/C2S3 showed very long IST and FST value (>100 min & 330 min).

**Figure 4:**
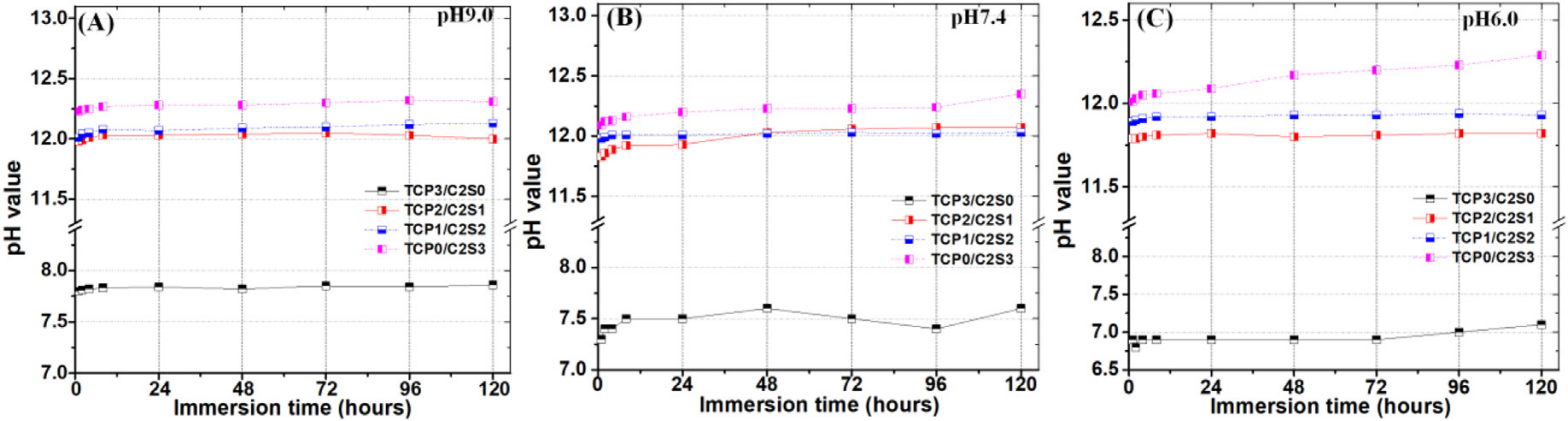
Changes in pH values of the immersion medium of the TCP/C2S cements prepared with different-pH value CaCl_2_ solutions.

Figure 4 showed the changes in pH value of immersion suspension with self-cured cements with different ratio of α-TCP and β-C2S. It was clear that the pH value of β-C2S-containing cements were equilibrated at ∼12.0. However, the pH value at each time point (2–120 h) for all of the suspension immersing the TCP3/C2S0 was maintained respectively at nearly 7.8, 7.5 and 6.9 with decreasing the pH value in the mixing solution from 9.0 to 6.0. However, the TCP2/C2S1 and TCP1/C2S2 composites showed an apparent alkaline feature (pH > 11.5).

### 3.4 Mechanical development and phasic transformation of the cements

**Figure 5:**
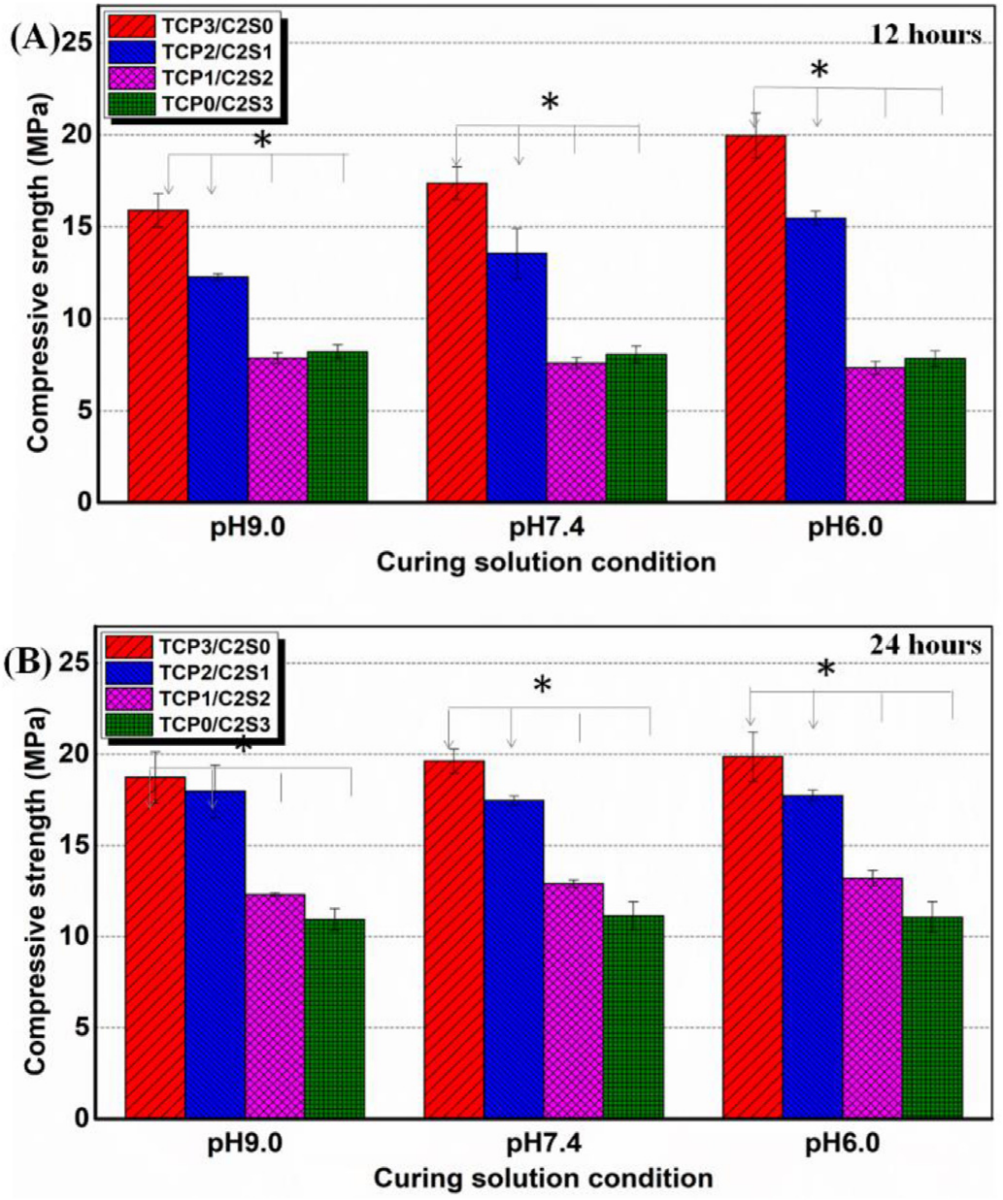
Changes in compressive strength of the TCP/C2S cements prepared with different-pH value CaCl_2_ solutions. ∗*p* < 0.05.

Figure 5 showed that all of four groups of cements set for 12 and 24 h exhibited appreciable compressive strength (>7 MPa & 10 MPa, respectively). The α-TCP component heavily influenced the compressive resistance of the cements. For instance, the strength of α-TCP-based cements (TCP3/C2S0, TCP2/C2S1) was ∼1.5–2.0-fold higher than the other two groups of cements after setting for 12 h. It was obvious that the high α-TCP content was favourable for enhancing the mechanical strength. However, the compressive strength of four groups of cements were increased with prolongation of setting time from 12 to 24 h, and that of the β-C2S-based cements (TCP1/C2S2, TCP0/C2S3) was increased by 50% between 12 and 24 h after setting.

**Figure 6:**
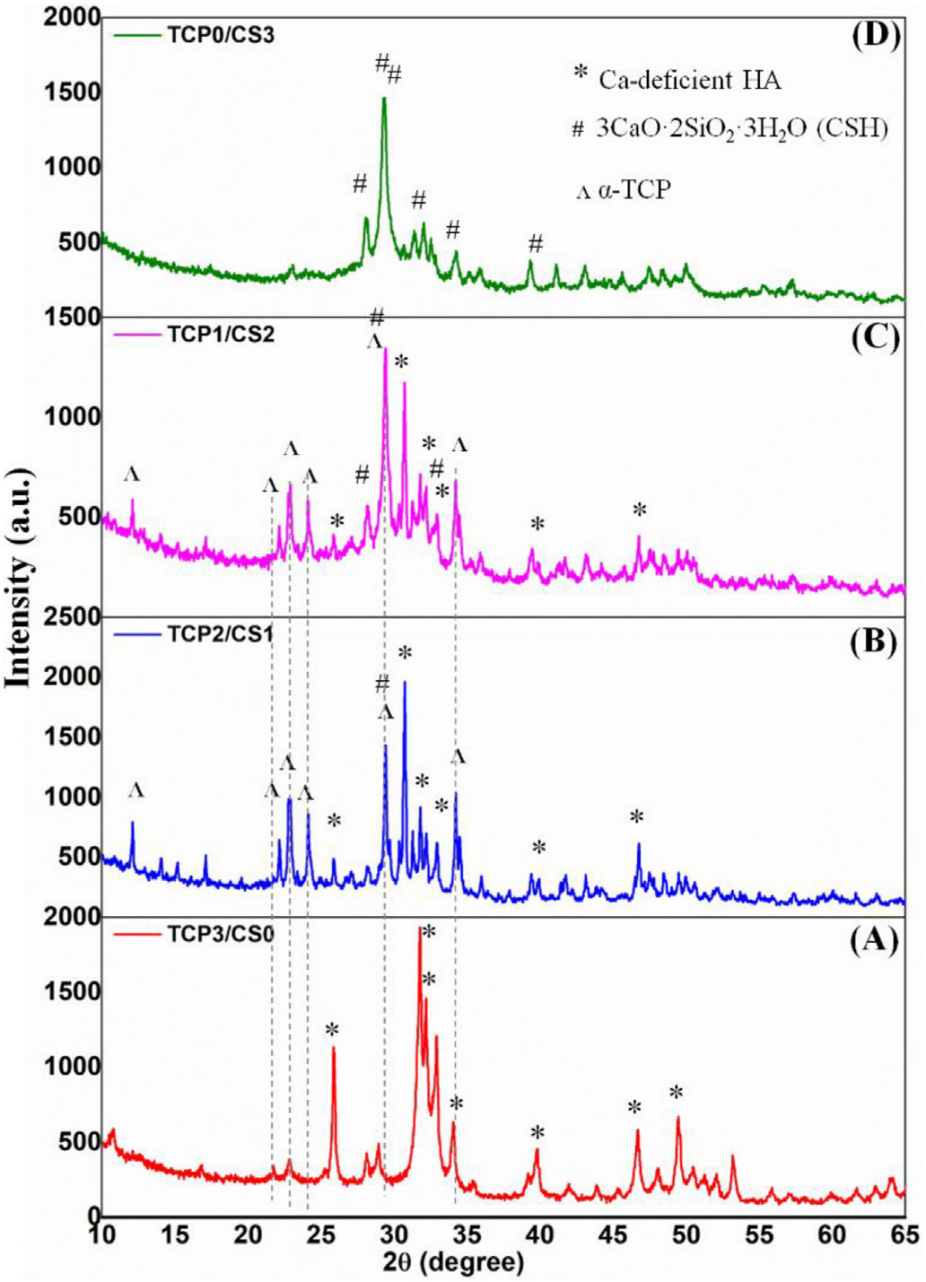
XRD patterns of the TCP/C2S cements prepared with pH 6.0 CaCl_2_ solution after curing for 3 days.

Figure 6 showed the XRD patterns of the cements prepared with pH 6.0 solution after setting for 120 h (5 days). It was worth noting that the TCP2/C2S1 and TCP1/C2S2 composite groups showed appreciable α-TCP residual after 5 days, but the other pure α-TCP (TCP3/C2S0) or β-C2S (TCP0/C2S3) cement has underwent a complete hydration reaction and the set products was only Ca-deficient hydroxyapatite (CDHA) and CSH (3CaO·2SiO_2_·3H_2_O), respectively.

### 3.5 Microstructure and re-mineralization potential of the cements in vitro

**Figure 7:**
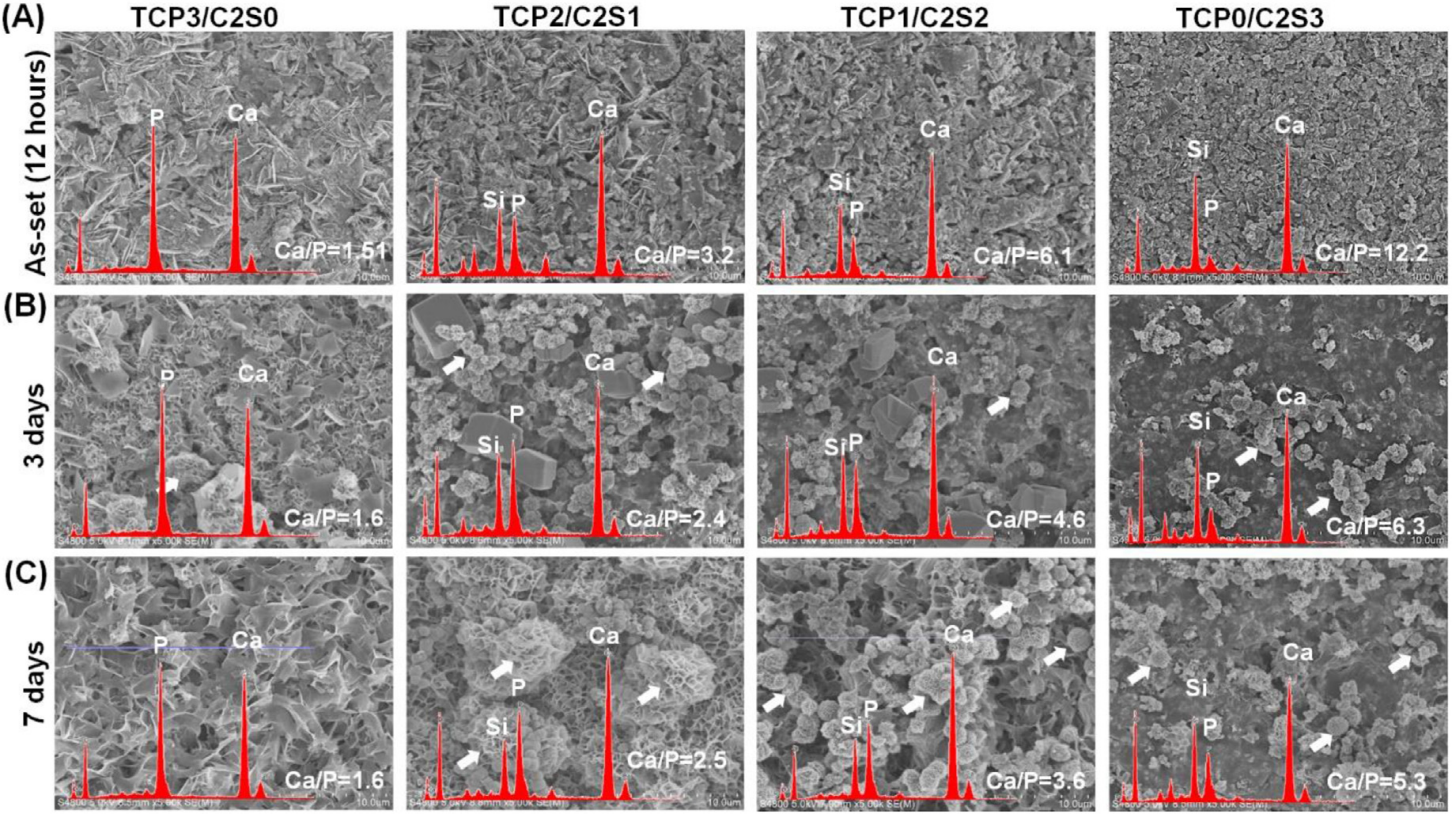
SEM images of the TCP/C2S cements prepared with pH 6.0 CaCl_2_ solution before and after immersion in artificial saliva for different time stages. Arrows showing the newly deposited calcium phosphate aggregates in the surface layer of the cement discs.

By the end of the primary self-setting reaction (12 h), the SEM images showed that the (TCP3/C2S0 and TCP0/C2S3) have been quickly transformed to blade- or aggregate-like CaP and CSH, respectively (Figure 7A). However, the biphasic composites in the fracture surface grew as a mass of denser aggregation state (Not shown). The spherical apatite-like aggregates could be observed from the β-C2S-containing cement samples after 3 days (Figure 7B), and the aggregates grew from 3 to 7 days (Figure 7C). The face-scanning EDX analysis confirmed that the Ca/P ratio in the surface layer was decreased over time and the peak of silicon was significantly decayed on the TCP1/C2S2 and TCP0/C2S3 samples.

**Figure 8:**
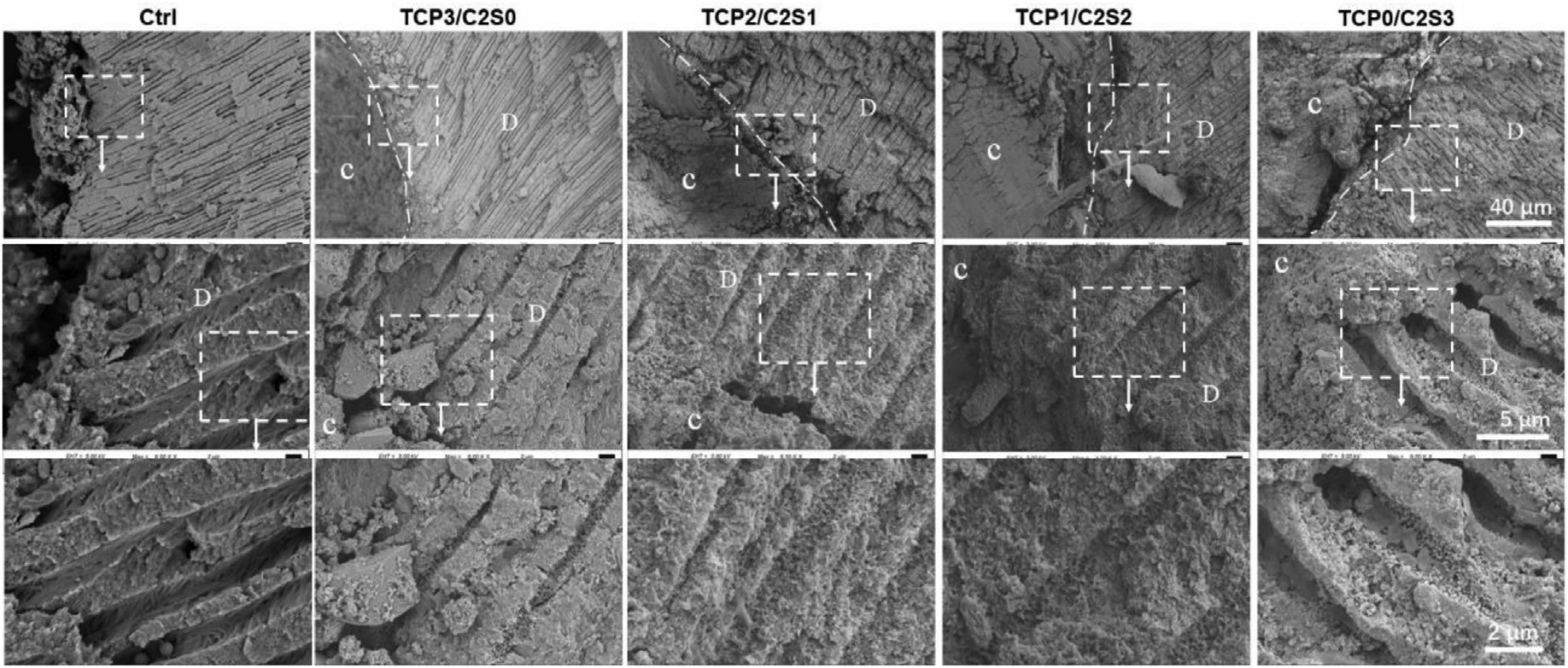
SEM micrographs of the TCP/C2S cements prepared with CaCl_2_ solution (pH 6.0) after immersion in artificial saliva for 5 days. The dotted line representing the interface between canal and filled cement; the dashed rectangles representing the selected area for high-magnification SEM observation; C: cement; D: dentine.

The SEM observation (Figure 8) confirmed that the paste was readily perfused into the apical root canal cavity and integrated with the side wall after immersion in simulated saliva for 5 days. The high-magnification SEM micrographs indicated that new apatite-like crystal clusters in several microns were deposited in the dentine tubules of the teeth filled with the cement pastes. However, the dentine tubules of the teeth free of filler (Ctrl) showed no apatite-like deposits after immersion for 5 days.

**Figure 9:**
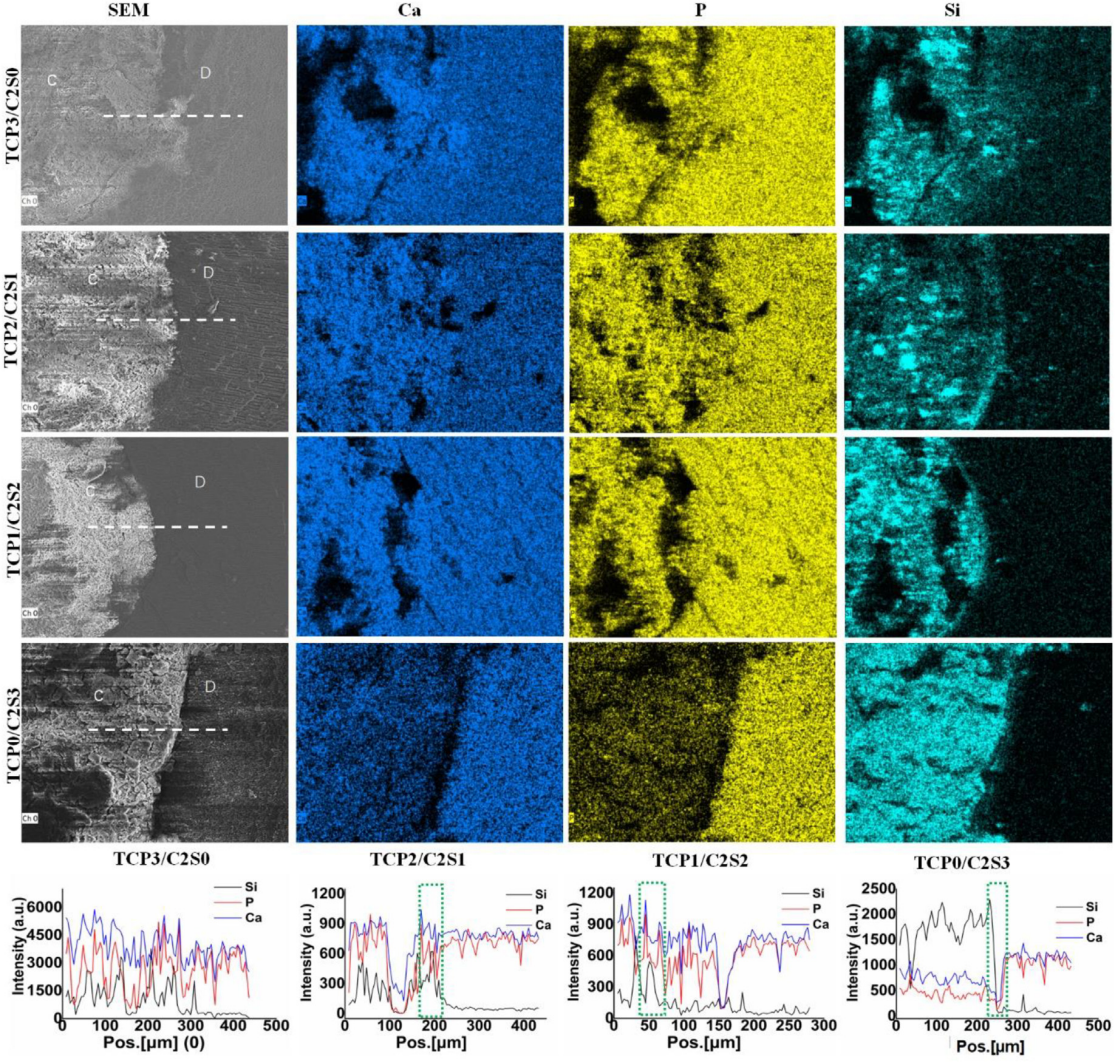
SEM observation and face/line-scanning EDS images of the TCP/C2S cements prepared with pH 6.0 CaCl_2_ solution after immersion in artificial saliva for 5 days. The line-scanning EDS analysis was carried out along the dotted-line in the SEM images; the dotted-line box in EDS images showing the cement-dentine interface zone. C: cement; D: dentine.

The cross-sectional element distribution in the interface region between self-cured cement and root canal wall was identified by face-scanning EDS analysis (Figure 9). It was revealed that there was a clear fluctuation of the Si peaks at the cement-dentine interface filled with β-C2S-containing cement samples. However, the fluctuation of Ca and P peaks could not be readily distinguished from the specimens filled with α-TCP-containing cements, because the cement and dentine both contained appreciable calcium ions and phosphate groups.

### 3.6 Anti-leakage potential of the pastes in vitro

**Figure 10:**
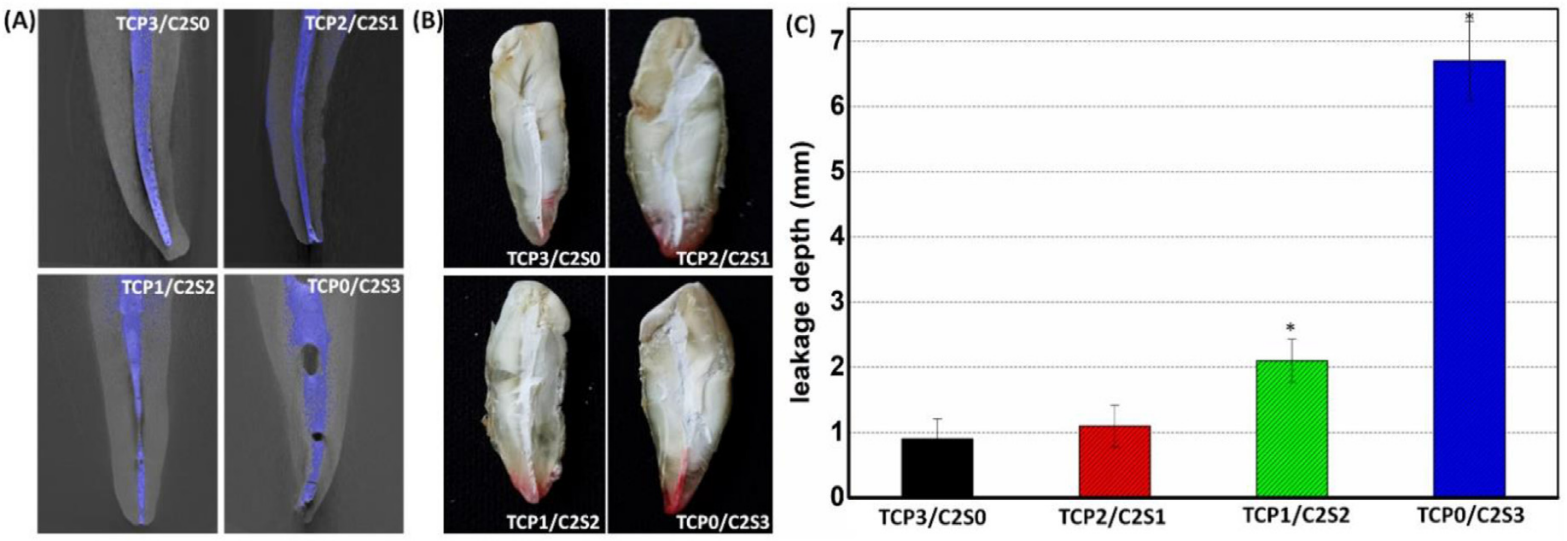
Cross-sectional μCT reconstruction (A) and micro-leakage evaluation (B, C) of the TCP/C2S cements prepared with pH 6.0 CaCl_2_ solution in root canal. ∗*p* < 0.05.

As can be seen from Figure 10A, the μCT image showed that all of the longitudinally sectioned root canal fillers with TCP/C2S cements had different adaptation to the canal wall, without gaps or voids, and afforded tighter adaptation for the paste containing low β-C2S component (i.e. TCP3/C2S0, TCP2/C2S1). With regard to adaptation to the root canal wall, there was no significant difference in the TCP3/C2S0 and TCP2/C2S1 groups. However, the β-C2S-based cements exhibited some different degree of shrinkage and deficient filling.

The ex vivo apical microleakage was measured in dye solution. According to the optical observation (Figure 10B), the microleakage distance (Rhodamine B dye penetration) of the filling cements was increased when the β-C2S content was increased. The TCP3/C2S0 and TCP2/C2S1 groups showed very limited microleakage, but the other two groups indicated appreciable microleakage. The pure β-C2S group could not overcome the risk of microleakage. According to the quantitative analysis (Figure 10C), the microleakage distances in the α-TCP-based cement groups were in the range of 0.9–1.2 mm, indicating a significant difference (*p* < 0.05) in comparison with the β-C2S-based cement groups (>2.0 mm), implying high α-TCP content may contribute on anti-microleakage.

## 4 Discussion

Modern concept of dentistry has changed since last few decades and the dentists continue to strive to conserve teeth, and this change in trend has been due to the realization of the fact that natural teeth function more efficiently than any artificial replacement according to multidisciplinary development [33]. Indeed, there are numerous situations where the completely physical sealing of root canal alone does not achieve the aim of optimal treatment. In such cases a multiple functional filling materials with high antibacterial and excellent re-mineralization potentials and fast setting reaction are a more reasonable alternative.

In our study, one new design focusing on the integration of fast curing property, HA mineralization, and anti-microleakage was reported. Some key features were significantly improved by developing bioactive TCP/C2S composite pastes as root canal filling materials. The biphasic composite cements prepared with different liquid pH values could reach an excellent physicochemical and biological performances, inducing short setting time, high alkaline level, apatite re-mineralization and resisting microleakage, while keeping the advantages of the biphasic composites.

The phase compositions based on XRD analysis show that the free CaO phase could be determined in the sol-gel-derived β-C2S powders. The quantitative data indicates that there is appreciable free CaO content (>5%) in the β-C2S powders. In this aspect, our previous studies have indicated that the Ca/Si ratio in the reactant systems are directly related with the free CaO content in the β-C2S powders [23]. The conventional Ca/Si ratio of 2.0 in the TEOS-hydrolyzed sol medium leads to very high free CaO content (>12%). This high CaO content may be attributed to the much faster polycondensation reaction of TEOS in strong acid medium, when comparing with the colloidal SiO_2_. In contrast, the silica colloid could maintain a mild hydrolysis reaction and thus the free CaO can be easily controlled [23, 34]. Our previous studies have also indicated that the free CaO in Ca-silicate powders is thought to be beneficial for reinforcing the alkaline level of the paste, and thus its antibacterial potential could be enhanced [23].

It is well agreed the setting time and injectability are two of most clinically relevant factors in root canal filling. Too long setting duration will cause clinical problems due to the cement's instability to maintain shape and sealability. In general, the powder composition, particle size, liquid phase and the L/P ratio play important roles in the self-setting efficacy [35, 36]. On the other hand, all the self-setting CaP formulations are made of an aqueous solution and fine (composite) powders, and the quick or slow dissolution of the initial calcium phosphate depends mainly on the chemical composition of powder and pH value of solution. In an aqueous environment, the dissolved Ca^2+^, HPO_4_^2-^ and/or PO_4_^3-^ ions form a supersaturated (very far away from the equilibrium) microenvironment and then precipitate as the calcium phosphate product(s) with lower dissolvability [37, 38]. Our study found that the pure α-TCP (i.e. TCP3/C2S0) and β-C2S (i.e. TCP0/C2S3) samples both showed longer setting time at the low L/S conditions (*L*/*P* = 0.7, 0.8), and as expected, the biphasic composites (TCP2/C2S1, TCP1/C2S2) pastes showed shorter *IST* value (Figure 2). Unfortunately, these composites still showed very high *FST* values (>160 min). It is reasonable to assume that the different particle size distributions of the different powder components in the TCP2/C2S1 and TCP1/C2S2 cements could be favourable for compensating the small intervals among the aggregates in the paste; thus, the hydrated granules could easily bond to each other and accelerate the self-setting process in the composite pastes. In fact, it is known that α-TCP is the most popular compound to produce self-setting calcium orthophosphate formulations. The hydration process (dissolution-polycondensation) of pure α-TCP in aqueous solution produces CDHA (Ca_9_(HPO_4_) (PO_4_)_5_(OH)) [39]:

(1) 3α-Ca_3_(PO_4_)_2_ + H_2_O = Ca_9_(HPO_4_) (PO_4_)_5_(OH)

The fast consumption of water during α-TCP hydration reaction in the early stage would lead to decrease of L/S ratio in the TCP2/C2S1 and TCP1/C2S2 cement pastes. Therefore, the different particle size distribution and hydration reaction speed between α-TCP and β-C2S both contribute a significant decrease of IST and FST.

As for the injectability of inorganic pastes for biomedical application, it is reported that gauge sizes of 10–16 would be useful for orthopedic procedures, whereas gauge sizes of 16–25 would be suitable for dental application [40, 41]. Thus, the pastes with different powder compositions and L/S ratio have been evaluated through the 20 G needle. It indicates that the composite powders at L/S ratio of 0.8 are readily injected through the 20 G needle. On the other hand, we found the setting efficiency of the TCP−C2S systems was heavily influenced by the pH level of the aqueous medium. The weak acidic aqueous medium (pH 6.0) is favourable for accelerating the self-curing reaction of the TCP2/C2S1 and TCP1/C2S2 pastes. The *IST* and *FST* values of composite pastes were only ∼20 and 130 min, respectively (Figure 3). These new findings demonstrate that the weak acidic medium is particularly advantageous to promote the early-stage hydraulic reaction and solidification behaviour of the composite systems. In fact, we also confirmed the different pH conditions in aqueous medium could not influence the alkaline nature of the TCP/C2S composites during immersion in Tris buffer (Figure 4). These results also demonstrate the weak alkaline nature of α-TCP [42]. In fact, it is reasonable to assume that the surface layer of α-TCP particles would undergo an increased dissolution reaction at the weak acidic medium, and then followed by a fast precipitation reaction to form CDHA [28]. Therefore, the weak acidic liquid could accelerate the self-curing process in the early stage. Meanwhile, it is expected that, both TCP2/C2S1 and TCP1/C2S2 cements show an apparent alkaline feature in buffer medium (pH > 11.5).

It is known that, in general, the higher is CaO content in β-C2S powders, the greater rate of pH increase can be recorded in the paste. Our previous studies have confirmed that the pH value of the immersion medium is directly related to the free CaO content in the β-C2S powder, and meanwhile the CaO content could be adjusted by Ca/Si ratio (from 2.0 to 1.4) in the sol reactant medium [23]. The increase of pH value in immersion solutions is mainly attributed to the hydroxide ion (OH^−^) release from the Ca(OH)_2_ by-products which are derived from the free CaO and β-C2S hydration reaction in aqueous medium:

(2) CaO + H_2_O→Ca(OH)_2_

(3) 2Ca_2_SiO_4_+4H_2_O→ 3CaO·2SiO_2_·3H_2_O + Ca(OH)_2_

Therefore, these hydration reactions from the β-C2S-based cements to CSH (3CaO·2SiO_2_·3H_2_O) may be thought to be high benefit for the early-stage antibacterial requirement in canal sealing treatment [43].

Moreover, it is agreed that the superfine α-TCP or β-C2S powder with aqueous solution in an appropriate ratio may form a paste that hardens by entanglement or aggregation of the crystal precipitated in the paste [44, 45]. In this regard, the choice of the mechanically mixed α-TCP−β-C2S systems is based on the setting and hydraulic reaction concerns that hydration reaction of α-TCP is faster than that of β-C2S in the early stage, so that it is mainly responsible for the initial cement setting and mechanical strength, while β-C2S contributes to the later hardens and apatite re-mineralization and continued antibacterial potential. Our experimental data also confirmed that the TCP−C2S cement systems may exhibit appreciable compressive resistance. It was found that the α-TCP component heavily influence the compressive resistance of the cements (Figure 5) and indeed the α-TCP component may contribute more mechanical strength. Totally, the TCP2/C2S1 had higher strength than the TCP1/C2S2, regardless of the pH values of CaCl_2_ solution. These experimental results indicate that compressive resistance of the α-TCP−β-C2S systems is mainly influenced by component content, but not the initial pH value of aqueous medium. On the other hand, it is very interesting that the presence of β-C2S component may retard the complete phase transformation of α-TCP in the early-stage self-curing process (Figure 6). This new phenomenon may be explained by the fact that, although the weak acidic solution can readily accelerate the hydration reaction of α-TCP in the early several hours, the fast consumption of water during phase transformation of α-TCP, free CaO, and β-C2S may lead to decrease of the free water among the powder granules. Thus, it is reasonable to consider that the hydration reaction in the core region of α-TCP granules would be inhibited due to absence of free water permeation.

Moreover, SEM observation may evaluate the interface region between the cement and root canal system [46]. It is known that HA re-mineralization of root canal filling materials benefits the integration with dentine wall and tublues [47, 48], and especially the newly deposited HA along the dentine tubules may even enhance anti-leakage and tooth hypersensitive resistance [49]. In our study, the spherical apatite-like aggregates could be easily observed on the β-C2S-rich cements. The SEM observation also confirmed that the HA-like re-mineralization could occur in the root canal-filled teeth during immersion in simulated saliva (Figures 8 and 9). The face-scanning EDX analysis also indicated the breaking peaks of P or Si at the interface region containing composite cements. Unfortunately, the control group (without filling cements) could not induce HA-like products in simulated saliva. These results suggest that the composite cements possess good HA-mineralization ability and exhibit expected complete filling potential in the apical root canal tubules.

It is well agreed that the μCT reconstruction and ex vivo apical microleakage are both favourable for approach to demonstrate the voids associated with different root canal fillers [50]. Our μCT reconstructed images indicate the α-TCP-rich samples may fill completely the root canal with good adaptation to the canal wall. In contrast, the β-C2S-based cements exhibited more shrinkage and deficient filling, and thus showed very appropriable microleakage and Rhodamine B dye penetration. This suggests that more α-TCP content in the composite pastes would be beneficial for anti-microleakage potential.

Finally, it is known that, to overcome the shortcoming of conventional Ca-silicate-based cements under a variety of clinical conditions, it is critical to develop novel strategies for endowing multiple functions to achieve a synergistic therapy. The present study summarized our effort to prepare highly bioactive filling cements composed of α-TCP−β-C2S biphasic composites. In order to achieve this objective, the rapid setting α-TCP component was used for adjusting the curing time and anti-microleakage of the β-C2S-containing root canal fillers to guarantee re-mineralization bioactivity and appropriate antibacterial potential, without compromising its sealability and biological properties. It is reasonable to consider that a layer of HA readily re-mineralize over the composite biomaterial that may fill the surface voids and even significantly attenuates or completely reverses bio-dissolution in the early stage. Accordingly, the excellent apatite mineralization potential of the α-TCP−β-C2S cement systems is of high benefit to improve chemical bonding between the material and the dentinal, and thus to avoid the potential detrimental effects.

## 5 Conclusion

In summary, this study has confirmed the advantage of β-C2S/α-TCP composites and the new fast curing, bioactive root canal fillers were developed. The adjustment of component contents in the cement pastes could tune the pH level (antibacterial potential), and addition of α-TCP in the β-C2S-containing paste has a considerable effect on the setting time, mechanical properties and re-mineralization ability, which are beneficial for hydraulic fillers in comparison to the pure β-C2S. Based on such simple composition characteristic, the α-TCP−β-C2S cement systems (e.g. TCP2/C2S1) would match the optimal criteria in a variety of applications such as root end closure, repair of root perforations, capping of dental pulp tissues, and even promoting original tissue regeneration.

## Declarations

### Author contribution statement

Youyang Zheng, Xianyan Yang, Feng Zhang, and Zhongru Gou: Conceived and designed the experiments; Wrote the paper.

Shuxin Liu, Siqi Bao, Yuyue Xu, and Yunyi Wang: Performed the experiments; Analyzed and interpreted the data.

### Funding statement

Dr. Youyang Zheng and Dr. Feng Zhang were supported by the Zhejiang Provincial Basic Research for Public Welfare Funds [LGF20H140005, LGF20H140008].

Dr. Zhongru Gou was supported by the National Key Research and Development Program of China (2017YFE0117700) and Mingguang Mingjiu S&T Co. Fund.

### Data availability statement

Data included in article/supp. material/referenced in article.

### Declaration of interest's statement

The authors declare no conflict of interest.

### Additional information

No additional information is available for this paper.

## References

[1] Priyanka S.R., Veronica Dr (2013). A literature review of root-end filling materials. IOSR J. Dent. Med. Sci..

[2] Li G-h, Niu L.-N., Zhang W., Olsen M., De-Deus G., Eid A.A., Chen J-h, Pashley D.H., Tay F.R. (2014). Ability of new obturation materials to improve the seal of the root canal system – a review. Acta Biomater..

[3] Nixdorf D.R., Law A.S., Look J.O., Rindal D.B., Durand E.U., Kang W., Agee B.S., Fellows J.L., Gordan V.V., Gilbert G.H. (2012). Large-scale vlinical endodontic research in the national dental practice-based research network: study overview and methods. J. Endod..

[4] Hammarstrom L., Blomlof L.B., Feiglin B., Lindskog S.F. (1986). Effect of calcium hydroxide treatment on periodontal repair and root resorption. Endod. Dent. Traumatol..

[5] Jia L., Zhang X., Shi H., Li T., Lv B., Xie M. (2019). The clinical effectiveness of calcium hydroxide in root canal disinfection of primary teeth: a meta-analysis. Med. Sci. Mon. Int. Med. J. Exp. Clin. Res..

[6] Mohammadi Z., Dummer P.M.H. (2011). Properties and applications of calcium hydroxide in endodontics and dental traumatology. Int. Endod. J..

[7] Gomes B.P., Ferraz C.C., Garrido F.D., Rosalen P.L., Zaia A.A., Teixeira F.B., de Souza-Filho F.J. (2002). Microbial susceptibility to calcium hydroxide pastes and their vehicles. J. Endod..

[8] Vianna M.E., Gomes B.P., Sena N.T., Zaia A.A., Ferraz C.C., de Souza Filho F.J. (2005). In vitro evaluation of the susceptibility of endodontic pathogens to calcium hydroxide combined with different vehicles. Braz. Dent. J..

[9] Bakland L.K., Andreasen J.O. (2012). Will mineral trioxide aggregate replace calcium hydroxide in treating pulpal and periodontal healing complications subsequent to dental trauma?. Review.

[10] Dioguardi M., Alovisi M., Sovereto D., Troiano G., Malagnino G., Cosola M.D., Cazzolla A.P., Laino L., Muzio L.L. (2021). Sealing ability and microbial leakage of root-end filling materials: MTA versus epoxy resin: a systematic review and meta-analysis. Heliyon.

[11] Lofroth M., Ghasemimehr M., Falk A., von Steyern P.V. (2019). Bisphenol A in dental materials – existence, leakage and biological effects. Heliyon.

[12] Roberts H.W., Toth J.M., Berzins D.W., Charlton D.G. (2008). Mineral trioxide aggregate material use in endodontic treatment: a review of the literature. Dent. Mater..

[13] Saxena P., Gupta S.K., Newaskar V. (2013). Biocompatibility of root-end filling materials: recent update. Restor Dent Endod.

[14] Meschi N., Li X., Van Gorpa G., Camilleri J., Van Meerbeek B., Lambrechts P. (2019). Bioactivity potential of Portland cement in regenerative endodontic procedures: from clinic to lab. Dent. Mater..

[15] Prati C., Gandolfi M.G. (2015). Calcium silicate bioactive cements: biological perspectives and clinical applications. Dent. Mater..

[16] Nishad K.V., Komath M., Unnikrishnan G. (2019). Synthesis of strontium orthosilicate (Sr_2_SiO_4_) by sol-gel method for the use in endodontic cements to enhance bioactivity and radio-contrast. Mater. Res. Express.

[17] Esteban-Tejeda L., Cabal B., Torrecillas R., Prado C., Fernandez-Garcia E., López-Piriz R., Quintero F., Pou J., Penide J., Moya J.S. (2016). Antimicrobial activity of submicron glass fibres incorporated as a filler to a dental sealer. Biomed. Mater..

[18] Zhou Y., Xua C., Wang X., Dou Y., Huan Z., Chang J. (2018). Fast setting tricalcium silicate/magnesium phosphate premixed cement for root canal filling. Ceram. Int..

[19] Wang X., Sun H., Chang J. (2008). Characterization of Ca3SiO5/CaCl2 composite cement for dental application. Dent. Mater..

[20] Radwan M.M., Nagi S.M., El-Hamid H.K.A. (2019). Physico-mechanical characteristics of tri-calcium silicate pastes as dentin substitute and interface analysis in class II cavities: effect of CaCl_2_ and SBF solutions. Heliyon.

[21] Ding S.J., Shie M.Y., Wang C.Y. (2009). Novel fast-setting calcium silicate bone cements with high bioactivity and enhanced osteogenesis in vitro. J. Mater. Chem..

[22] Liu W., Wu C., Liu W., Zhai W., Chang J. (2013). The effect of plaster (CaSO_4_∙1/2H_2_O) on the compressive strength, self-setting property and in vitro bioactivity of silicate-based bone cement. J. Biomed. Mater. Res. Part B Applied Biomater..

[23] Yang X., Liu M., Zhao Y., Jia H., Xu S., Li X., Chen X., Zhang F., Gao C., Gou Z. (2014). Rational design and fabrication of a β-dicalcium silicate-based multifunctional cement potential for root canal filling treatment. J. Mater. Chem. B.

[24] Zhang F., Yang X., Zhuang C., Wang L., Gu X.H., Shen Z., Xu S., Gao C., Gou Z. (2016). Design and evaluation of multifunctional antibacterial ion-doped β-dicalcium silicate cements favorable for root canal sealing. RSC Adv..

[25] Dorozhkin S.V. (2009). Calcium orthophosphate cements and concretes. Materials.

[26] Abedi-Amin A., Darvizeh A., Luzi A., Mongiorgi R., Sauro S. (2014). Innovative light-curable calcium phosphate cements as retrograde filling material. Dent. Mater..

[27] Zima A., Czechowska J., Siek D., Olkowski R., Noga M., Lewandow-Szumieł M., Ślósarczyk A. (2017). How calcite and modified hydroxyapatite influence physicochemical properties and cytocompatibility of alpha-TCP based bone cements. J. Mater. Sci. Mater. Med..

[28] Carrodeguas R.G., Aza S De (2011). a-Tricalcium phosphate: synthesis, properties and biomedical applications. Acta Biomater..

[29] Haugen H.J., Basu P., Sukul M., Mano J.F., Reseland J.E. (2020). Injectable biomaterials for dental tissue regeneration. Int. J. Mol. Sci..

[30] Szurkowska K., Szeleszczuk Ł., Kolmas J. (2020). Effects of synthesis conditions on the formation of Si-substituted Aalpha tricalcium phosphates. Int. J. Mol. Sci..

[31] Lea F.M. (1981).

[32] Gou Z., Chang J., Zhai W., Wang J. (2005). Study on the self-setting property and the *in vitro* bioactivity of β-Ca_2_SiO_4_. J. Biomed. Mater. Res., Part B.

[33] Nassar H., Al-Dabbagh N., Aldabbagh R., Albahiti M., Jadu F.M., Qutob A., Mawardi H. (2020). Dental follow-up and maintenance index: the development of a novel multidisciplinary protocol. Heliyon.

[34] Gou Z., Chang J. (2004). Synthesis and in vitro bioactivity of dicalcium silicate powders. J. Eur. Ceram. Soc..

[35] Liu C., Shao H., Chen F., Zheng H. (2006). Rheological properties of concentrated aqueous injectable calcium phosphate cement slurry. Biomaterials.

[36] Burguera E.F., Xu H.H., Sun L. (2008). Injectable calcium phosphate cement: effects of powder-to-liquid ratio and needle size. J. Biomed. Mater. Res. Part B Appl Biomater.

[37] Lacout J., Mejdoubi E., Hamad M. (1996). Crystallization mechanisms of calcium orthophosphate cement for biological uses. J. Mater. Sci. Mater. Med..

[38] Son Y., Feng Z., Wang T. (2007). In situ study on the curing process of calcium phosphate bone cement. J. Mater. Sci. Mater. Med..

[39] Dorozhkin S.V. (2013). Self-setting calcium orthophosphate formulations. J. Funct. Biomater..

[40] Burguera E.F., Xu H.H.K., Sun L. (2008). Injectable calcium phosphate cement: effects of powder-to-liquid ratio and needle size. J. Biomed. Mater. Res. Part B: Appl Biomater.

[41] Komath M., Varma H.K. (2003). Development of a fully injectable calcium phosphate cement for orthopedic and dental applications. Bull. Mater. Sci..

[42] Lee J.-B., Park S.-J., Kim H.-H., Kwon Y.-S., Lee K.-W., Min K.-S. (2014). Physical properties and biological/odontogenic effects of an experimentally developed fast-setting α-tricalcium phosphate-based pulp capping material. BMC Oral Health.

[43] Janini A.C.P., Bombarda G.F., Pelepenko L.E., Marciano M.A. (2021). Antimicrobial activity of calcium silicate-based dental materials: a literature review. Antibiotics.

[44] Dorozhkin S.V. (2013). Self-Setting calcium orthophosphate formulations. J. Funct. Biomater..

[45] Zuleta F., Murciano A., Gehrke S.A., Maté-Sánchez de Val J.E., Calvo-Guirado J.L., De Aza P.N. (2017). A new biphasic dicalcium silicate bone cement implant. Materials.

[46] Camilleri J. (2011). Scanning electron microscopic evaluation of the material interface of adjacent layers of dental materials. Dent. Mater..

[47] Iftikhar S., Jahanzeb N., Saleem M., Rehman S., Matinlinna J.P., Khan A.S. (2021). The trends of dental biomaterials research and future directions: a mapping review. Saudi Dent J.

[48] Watson T.F., Atmeh A.R., Sajini S., Cook R.J., Festy F. (2014). Present and future of glass-ionomers and calcium-silicate cements as bioactive materials in dentistry: biophotonics-based interfacial analyses in health and disease. Dent. Mater..

[49] Primus C.M., Tay F.R., Niu L.-N. (2019). Bioactive Tri/dicalcium silicate cements for treatment of pulpal and periapical tissues. Acta Biomater..

[50] Moinzadeh A.T., Zerbst W., Boutsioukis C., Shemesh H., Zaslansky P. (2015). Porosity distribution in root canals filled with guttapercha and calcium silicate cement. Dent. Mater..

